# Diuretic Wine

**Published:** 1855-08

**Authors:** 


Diuretic Wine.—M. Granel suggests the following as a diuretic preparation, viz., squill sliced, and digitalis leaves bruised, each two drachms; best cinnamon three drachms; acetate of potassa half an ounce; Madeira wine a pint. Macerate during eight days and filter.

The dose is a table-spoonful in the morning.

The dose may be increased to four spoonsful a day: two in the morning and two in the evening, at least three hours after the last meal.—*Jour. of Pharm.*

